# Fitness benefits of trypsin proteinase inhibitor expression in *Nicotiana attenuata *are greater than their costs when plants are attacked.

**DOI:** 10.1186/1472-6785-4-11

**Published:** 2004-08-10

**Authors:** Jorge A Zavala, Ian T Baldwin

**Affiliations:** 1 Department of Molecular Ecology, Max Planck Institute for Chemical Ecology, Beutenberg Campus, Hans-Knöll Strasse 8, Jena 07745, Germany

## Abstract

**Background:**

The commonly invoked cost-benefit paradigm, central to most of functional biology, explains why one phenotype cannot be optimally fit in all environments; yet it is rarely tested. Trypsin proteinase inhibitors (TPIs) expression in *Nicotiana attenuata *is known to decrease plant fitness when plants compete with unattacked conspecifics that do not produce TPIs and also to decrease the performance of attacking herbivores.

**Results:**

In order to determine whether the putative benefits of TPI production outweigh its cost, we transformed *N. attenuata *to silence endogenous TPI production or restore it in a natural mutant that was unable to produce TPIs. We compared the lifetime seed production of *N. attenuata *genotypes of the same genetic background with low or no TPI to that of genotypes with high TPI levels on which *M. sexta *larvae were allowed to feed freely. Unattacked low TPI-producing genotypes produced more seed capsules than did plants with high TPI levels. Caterpillar attack reduced seed capsule production in all genotypes and reversed the pattern of seed capsule production among genotypes. *M. sexta *larvae attacking genotypes with high TPI activity consumed more TPI, less protein, and move later to the young leaves. Larval masses were negatively correlated (R^2 ^= 0.56) with seed capsule production per plant.

**Conclusions:**

Our results demonstrate that the fitness benefits of TPI production outweigh their costs in greenhouse conditions, when plants are attacked and that despite the ongoing evolutionary interactions between plant and herbivore, TPI-mediated decreases in *M. sexta *performance translates into a fitness benefit for the plant.

## Background

The cost-benefit paradigm is central to functional biology and to ecological and evolutionary theory because fitness costs and benefits associated with a trait determine its equilibrium value in a population. If the trait offers fitness benefits to the population rather than costs, then selection should lead to beneficial allele(s) being fixed, which reduces variability [1]. Alternatively, when the fitness benefit of the trait also has a cost, intermediate frequencies of the trait may be favored because the benefit varies [1-3]. For example, resistance against natural enemies has costs as well as obvious benefits for fitness, as has been shown in insect-parasite, insect-parasitoid, plant-pathogen and plant-insect systems [4-7].

Herbivores can reduce seed production and other correlates of plant fitness, and this reduction can result in natural selection for either constitutively expressed or inducible plant defenses [8-10]. Current theory predicts that one benefit of induced defenses is to allow a plant to optimize its allocation of limiting resources to defense, growth, and reproduction [9]. Although defenses might benefit plants in the presence of herbivores, plant resistance to herbivores can be costly in the absence of enemies, and inducible expression of resistance traits allow plants to forgo or, to pay the potential fitness cost of resistance traits when they are needed [3,5,11-14].

Evidence for the existence of resistance costs and benefits from studies using plant species with constitutive and inducible defenses is increasing [3,14-16]. Experiments on natural populations of plants as diverse as *Arabidopsis*, *Ipomea*, *Pastinaca *and *Trifolium *have provided evidence for costs [2,17-20]. These experiments typically use quantitative genetic approaches to determine whether, in the absence of enemies, fitness and resistance are inversely correlated. However, attribution of fitness consequences to the expression of a particular defense trait in an environment either with or without herbivory is difficult, because genes that control the expression of defensive traits may have pleiotropic effects [21]. Ideally, one should assess the costs and benefits of inducible defenses in plants that differ only in the expression of genes that control (induced) resistance but are otherwise genetically identical [15]. Transformation technology provides a means of manipulating traits with unparalleled precision. Although the benefits of plant traits that provide resistance against herbivores are expected to equal or exceed their cost when the system is at evolutionary equilibrium [22-25], very few direct tests have been done. While costs and putative benefits of defense traits have been studied in separate experiments, their currencies are usually not comparable (i.e., plant fitness for the cost; herbivore performance for the benefits). Tests of the cost-benefit model using the same currency are few [5] and these studies do not consider the heterogeneity of the plant.

Ecological interactions can be viewed as the net outcome of a series of cost-benefit optimizations in which both players respond to the variability in each others' defense traits. For example, there is enormous within-plant heterogeneity of defensive secondary metabolites. This heterogeneity could motivate within-plant movement of herbivores, so that they eat leaves of low fitness value rather than leaves of high fitness value, or it could motivate herbivores to move off plants and onto neighboring competitors [26,27]. Herbivores, in turn, can both readjust their metabolism to cope with the secondary metabolites as well as adjust their feeding positions to maximize their performance [27-29]. We present here a cost-benefit analysis of a plant-insect interaction in which the costs and benefits of a defensive protein are evaluated in the currency of plant fitness.

*Nicotiana attenuata *[Torr. Ex Wats. (synonymous with *Nicotiana torreyana *Nelson and Macbr.)], a postfire annual native tobacco inhabiting the Great Basin Desert, has a number of well-described herbivore-induced direct and indirect defenses [30], which increase the fitness of plants under attack in natural populations [5,31]. Trypsin proteinase inhibitors (TPI) play an important defensive role in addition to nicotine [30]. We isolated cDNA from *N. attenuata *that coded for a TPI precursor belonging to the potato PI-II family with a 7-repeat TPI domain. The normal constitutive expression of this gene increases 4-fold after herbivore attack [32,33].

The elicitation of TPI expression in *N. attenuata *varies with ontogeny and leaf age [34], as is true for nicotine [35] and volatile emissions [36]. The within-plant pattern of systemic TPI induction at the rosette stage of growth suggests that the signal(s) triggering remote TPI induction follows a source-sink relationship; regardless of ontogenetic stage, if young sink leaves are damaged, TPI levels increase only in the attacked leaf, while older leaves are less sensitive to leaf damage and produce a less intense response in the attacked leaf, the systemic responses in young leaves is dramatic [34]. The spatial and temporal variability in *N. attenuata*'s ability to deploy certain defenses against herbivores can be correlated with the relative fitness values of leaves growing at particular nodes. Removal of young and mature leaves at the elongation stage in *N. sylvestris *had a greater negative effect on fitness than did the removal of old leaves, but not at either the rosette or flowering stages, demonstrating the different fitness values of leaves growing at different nodes on a plant. Damage to younger leaves increases nicotine contents more than damage to older leaves does, suggesting that defense allocation is proportional to the fitness value of the tissue, as predicted by Optimal Defense (OD) theory [10,23,35,37].

**Figure 1:**
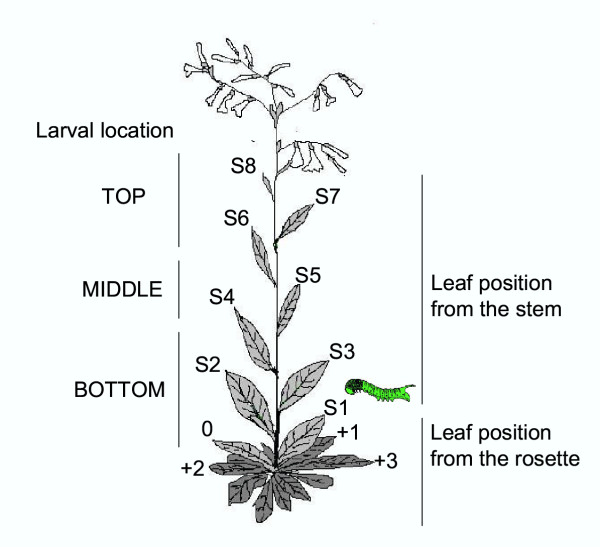
Sketch of *Nicotiana attenuata *plant showing different leaf positions on either the rosette or the stem [38] and larval location. Larva depicts the leaf growing at node S1 on which a single *M. sexta *neonate was placed.

*Manduca sexta*, a specialized lepidopteran herbivore, prefers elongating *N. attenuata *plants to rosette-stage plants for oviposition and places eggs on leaves in the middle section of the stem (from S1 to S3; Figure 1; [38]). TPIs of *N. attenuata *leaves reduce the growth of *M. sexta *larvae [32,33]. However, insects may adapt to high TPI levels, replacing the inhibited trypsin with the secretion of trypsins that are insensitive to the particular TPIs of the diet [29,39]. Intra-plant movement of the first instar larvae is very rare but common in the second-to fourth-larval instars [38]. Larger instars are heavier and more difficult to handle by insect predators, and also better able to defend themselves against an attack in their natural environment by vigorous movement and regurgitation (A. Kessler, personal communication). Larvae are particularly sensitive to jasmonate-induced defenses during the third instar (approx. 11 days after hatching), and can be motivated to move between adjacently growing plants [26] by the plant's induced defense. When *M. sexta *larvae were placed on MeJA-induced plants, larvae left the induced plants 1–3 days earlier than did larvae placed on uninduced plants, which dramatically reduced the leaf area consumed and larval weight gain [40].

TPI expression in *N. attenuata *is known to decrease lifetime seed production in unattacked but competing plants [32] and to decrease *M. sexta *performance in attacked plants [32]. Whether the TPI-mediated decrease in herbivore performance translates into a fitness benefit for the plant is unknown. In other systems, plants expressing high PI levels caused herbivores to grow more slowly, but they compensated by eating more tissue, a potential fitness detriment for the plant [41]. Here we provide a critical test of whether the fitness benefits of TPI expression outweigh their costs.

We compare lifetime seed production of *N. attenuata *genotypes with either low or no TPI production to that of TPI-producing genotypes on which *M. sexta *larvae were allowed to feed freely for 11 days. TPI and protein content were measured in all genotypes at all leaf positions. *M. sexta *larval mass and movement were recorded, and we calculated and simulated the amount of TPI and protein consumed by the larvae from the larval movement and the TPI and protein concentration at each leaf position from each genotype. We used two independently transformed *N. attenuata *lines in which the expression of the *pi *gene was down-regulated by antisense expression of a 175 bp fragment of the *N. attenuata pi *gene (AS –, AS-), and untransformed wildtype plants (WT) of the same genetic background (an inbred line collected from Utah). In addition, we used a natural *N. attenuata *genotype collected from Arizona, which has a mutation in the endogenous 7-domain *pi *gene and does not produce *pi *transcripts or TPI activity (A). We transformed this genotype with the full-length cDNA of the 7-domain *pi *gene in a sense orientation under control of a constitutive promotor (S++), so that after 11 days of caterpillar attack it produced TPIs at 74 % of the level found in the stem leaves of the wildtype Utah genotype. Our analysis demonstrates that the fitness benefits of TPIs production outweigh their cost when plants are attacked.

## Results

### Spatial and temporal distribution of plant TPI/protein contents

**Figure 2:**
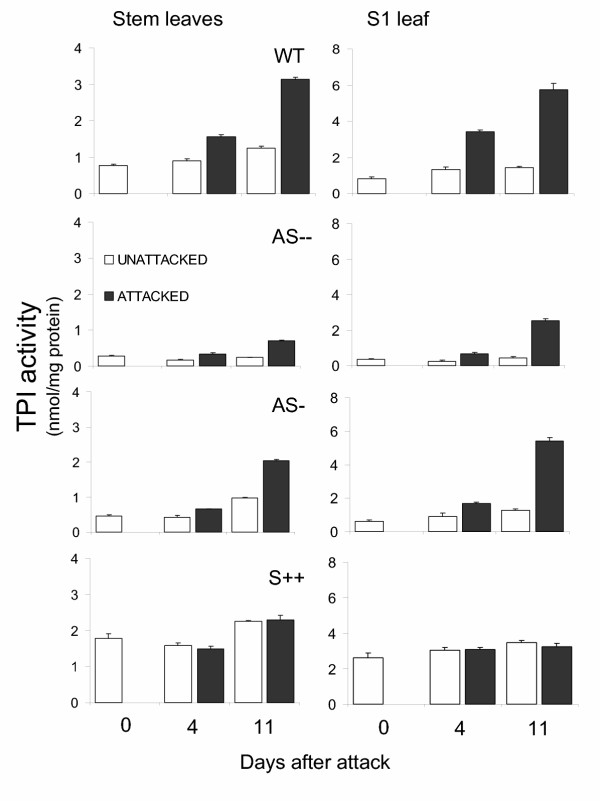
TPI activity (mean ± SEM) from stem leaves and the leaf growing at node S1 of untransformed wild type *Nicotiana attenuata *plants of the Utah genotype (WT); two homozygous T_3 _independently transformed lines of the Utah genotype that had been transformed with a construct containing a 175 bp *pi *gene fragment in an antisense orientation (AS –, AS-); plants of a homozygous T_3 _transformed line of the Arizona genotype transformed with a construct containing the full-length *pi *gene in a sense (S++) orientation before attack (day 0); and either unattacked or attacked by *M. sexta *larvae 4 and 11 d after neonates started to feed on the leaf at S1 position. Thin bars indicate ± SEM.

**Figure 3:**
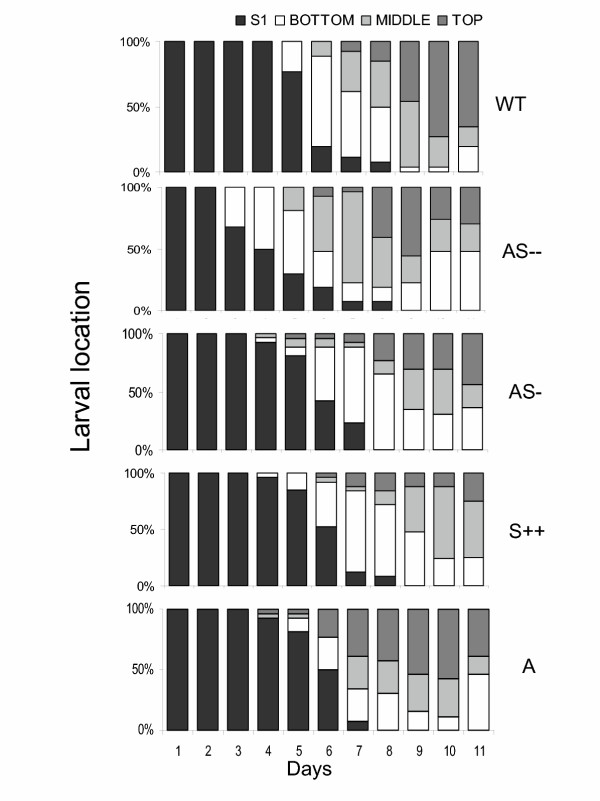
Relative number of *M. sexta *larvae on different plant locations on WT, AS –, AS-, S++, and Arizona (A) genotypes during 11 days on leaves growing at node S1, or the bottom, middle or top part of the plant (Figure 1). A single *M. sexta *neonate was placed on the leaf growing at node S1 and larval movement was monitored.

**Figure 4:**
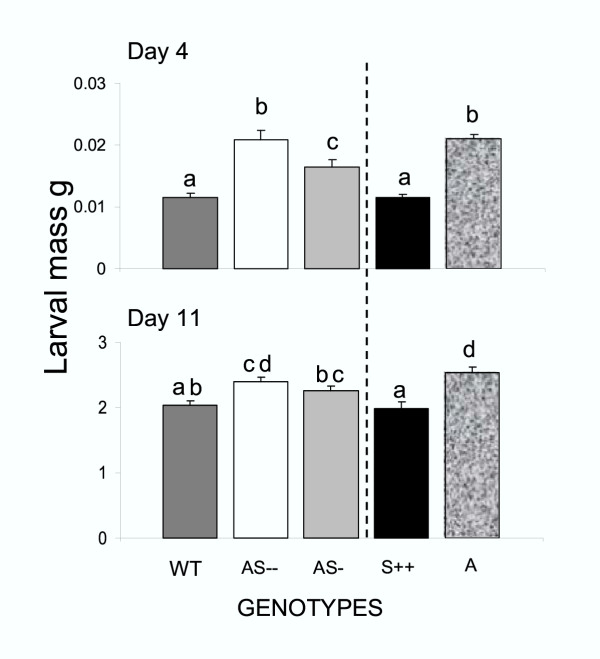
*M. sexta *mass (mean ± SEM) at 4 and 11d after neonates started to feed on leaves at S1 position (elongation stage) from WT, AS –, AS-, S++, and Arizona (A) genotypes. Bars with the same letter are not significantly different at P < 0.05 determined by one-way ANOVA. Thin bars indicate ± SEM.

In order to determine the effect of caterpillar attack on TPI activity, measurements were made from all rosette and stem leaves before, and 4 and 11 d after larvae started to feed on S1 leaves (Fig. 1) from transformed (AS –, AS-, and S++) and untransformed (WT and A) genotypes (Fig. 2 and Fig. 1-4/Appendix 1 [see Additional file 1 ]). All genotypes had high within-plant heterogeneity of TPI activity and protein contents. Constitutive TPI levels in all genotypes on day 0 (before larvae started to feed) were higher in rosette leaves than in stem leaves (F_1,70-AS – _= 217.13; P <0.0001; F_1,70-AS- _= 357.76; P <0.0001; F_1,70-WT _= 209.8; P <0.0001; F_1,70-S++ _= 4.27; P = 0.04), while protein content showed the opposite pattern, with higher levels in stem leaves than in rosette leaves (F_1,70-AS – _= 331.8; P <0.0001; F_1,70-AS- _= 256.6; P <0.0001; F_1,70-WT _= 289.6; P <0.0001; F_1,70-S++ _= 1.87.1; P <0.0001; Fig. 1-4/Appendix 1) which persisted through the samplings performed on day 4 and 11 (data not shown). A-genotype plants had a similar pattern in protein content (data not shown; F_1,70-A _= 245.5; P <0.0001). Caterpillar attack increased levels and within-plant heterogeneity of TPI activity. Larval damage to WT plants increased TPI activity 2.5-fold in S1 leaves (F_1,14 _= 197.0; P <0.0001) and 1.7-fold in unattacked (F_1,110 _= 17.3; P <0.0001) stem leaves, and did not alter TPI activity in older rosette leaves 4 d after neonates started to feed (F_1,62 _= 0.04; P = 0.8; Fig. 2 and Fig. 1/Appendix 1). By day 11, TPI activity had increased in WT S1 leaves 4-fold (F_1,22 _= 183.3; P <0.0001), 2.5-fold in the stem leaves (S avg; F_1,334 _= 337.0; P <0.0001; Fig. 2), and also marginally (1.1-fold) on the rosette leaves (F_1,94 _= 8.6; P <0.004; Fig. 1/Appendix 1).

Levels and within-plant heterogeneity of TPI activity were either intermediate or low in AS compared to WT plants after larval damage. After 4 days of caterpillar attack, TPI levels in AS – and AS-genotypes were 60 % and 40 % lower than those of unattacked WT (F_1,190-AS–total _= 62.4; P < 0.0001; F_1,190-AS-total _= 23.4; P < 0.0001; Figs 2 and 3/Appendix 1). Caterpillar attack increased TPI activity 2.4-fold in S1 leaves in AS plants, attaining values that were 19% and 48% in AS – and AS-plants, respectively of that in attacked WT plants (F_1,14-AS–S1 _= 630.3; P < 0.0001; F_1,14-AS-S1 _= 193.2; P < 0.0001; Fig. 1); TPI levels in the stem leaves were 37% and 55% of that found in induced WT plants (F_1,190-AS– _= 89.8; P < 0.0001; F_1,190-AS- _= 42.1; P < 0.0001; Fig. 2). By day 11, TPI levels in stem leaves were 22% in AS – and 65% in AS-of the WT levels (F_2,501_= 225.5; P < 0.0001; Fig. 2).

As expected, caterpillar attack did not affect either levels or within-plant heterogeneity of TPI activity of S++ plants. Compared to the constitutive levels of TPI activity in the WT, levels in S++ plants on day 4 were 30% higher (F_1,190-total _= 23.8; P < 0.0001; Fig. 4/Appendix 1). Caterpillar attack did not alter TPI activity in S++ plants (F_1,430-total _= 0.1; P = 0.06; Fig. 4/Appendix 1) which remained at approximately 90% of the induced WT plants in the S1 leaf and 1.1-fold at the plant level (averaged across all measured leaf positions; F_1,14-S++-S1 _= 3.8; P = 0.06; F_1,190-total _= 0.8; P = 0.3; Fig. 2). By day 11 d, TPI activity in S++ plants were 56% in the S1 leaf (F_1,22 _= 48.8; P < 0.0001) and 74% in stem leaves of the induced WT levels (F_1,430 _= 72.3; P < 0.0001; Fig. 2). As expected, the untransformed A genotype showed no TPI activity even after caterpillars had fed on the plant for 4 or 11 d. Protein levels did not differ significantly among genotypes. In summary, TPI levels in AS – and AS-genotypes in S1 and stem leaves were lower than in WT plants without differences in protein contents. Absolute TPI levels were substantially lower in the AS genotypes after caterpillar attack and S++ genotype produced TPI levels that were 74% of the activity found in induced WT plants.

### Within-plant movement of M. sexta larvae

To determine the effect of TPI on within-plant movement of *M. sexta *larvae, we measured the position of each larva on each plant daily. Caterpillars on low TPI genotypes left the S1 leaf and moved to the BOTTOM of the plant earlier than those feeding on high TPI genotypes (Fig. 3). While larvae on WT plants started to move from the S1 leaf to the BOTTOM of the plant after 5 d, larvae on AS – plants started to move 2 d earlier (day 3), and those that fed on AS-plants started to move 1 d earlier (day 4; Fig. 3). This early larval movement resulted in more larvae on the TOP and MIDDLE parts of plants from the AS – genotype (51 %, 77 %, and 80%) than on WT plants (11 %, 37 %, and 49 %) during subsequent days (days 6–8; Mann-Whitney *U*-test; P <0.0001; Fig. 3). If caterpillars prefer to feed on leaves with low TPI levels, then we would expect to have higher defoliation levels of plants with either no or low TPI compared to those with high TPI levels, increasing the number of caterpillars on the BOTTOM after some days. By day 11, 65 % of the larvae on WT plants were on the top and 19 % were on the BOTTOM, while on AS – plants, 30 % were on the TOP and 48 % on the BOTTOM, and on AS-plants 44 % were on the TOP and 36 % on the BOTTOM (Mann-Whitney *U*-test; P_WT-AS–TOP _= 0.001; P_WT-AS–BOTTOM _= 0.03; P_WT-AS-TOP _= 0.01; P_WT-AS-BOTTOM _= 0.3; Fig. 3).

Similar movement patterns were found in larvae on S++ and A genotypes. Larvae on A plants moved earlier (day 4) from the leaf at node S1 and toward the MIDDLE and TOP of the plant compared to larvae on S++ plants (Fig. 3). This earlier movement was reflected in the number of larvae on the MIDDLE and TOP of the plant from day 6 to 9 with a greater percentage in A (23 %, 65 %, 69 %, and 84 %) than in S++ (8 %, 16 %, 28 %, and 56 %) genotypes (Mann-Whitney *U*-test; P <0.0001; Fig. 3). On day 11, there were no differences in the number of caterpillars between A and S++ plants at the BOTTOM and at TOP of the plant (Mann-Whitney *U*-test; P_A-S++TOP _= 0.35; P_A-S++BOTTOM _= 0.1; Fig. 3). In summary, lighter caterpillars moved later than heavier caterpillars upward within the plant during the first days, and on day 11 they moved later to the BOTTOM of the plant.

### Calculated and simulated TPI and protein consumed by M. sexta larvae

**Figure 5:**
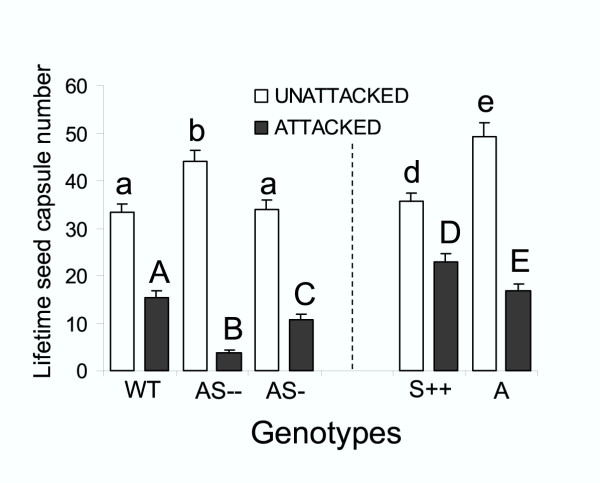
Mean capsule number from WT, AS –, AS-, S++, and A genotypes that were either unattacked or attacked by *Manduca sexta *larvae for 11 days. Bars with the same letter within a group are not significantly different at P < 0.01 determined by one-way ANOVA. Thin bars indicate ± SEM.

We calculated the amount of TPI and protein consumed by *M. sexta *larvae during the first, second, and third instars from each larvae's instar-specific feeding site, the concentration of leaf protein and TPI at the feeding site, and the instar-specific consumption from literature values (Tables 1a and b/Appendix 1). Plant genotype strongly influenced the calculated amount of TPI and protein consumed. Calculated total TPI and TPI consumed during the first, second and third instars were the highest for larvae on WT (16.7 g total) and the lowest for larvae on AS – (2.9 g total) plants (F_2,76-Total _= 888.6; P < 0.0001; F_2,76-First _= 28419.3; P < 0.0001; F_2,76-Second _= 442.8; P < 0.0001; F_2,76-Third _= 671.2; P < 0.0001; Fig. 5/Appendix 1). During the second instar, larvae on AS – plants consumed the highest calculated amount of protein (1.5 g), larvae on WT plants, the lowest (0.8 g), but no differences were found between genotypes during the first and second instars (F_2,76-First _= 1.8; P = 0.1; F_2,76-Second _= 87.14; P < 0.0001; F_2,76-Third _= 2.1; P = 0.1; Fig. 5/Appendix 1). As expected, the calculated total amount of protein consumed was higher on larvae fed on AS – (7.0 g) than those fed on either AS-(6.6 g) or WT (6.3 g) genotypes (F_2,76-Total _= 11.6; P < 0.0001; Fig. 5/Appendix 1). Larval mass of caterpillars fed on WT, AS –, and AS-genotypes was affected by the amount of TPI (F_2,76-11d _= 10.2; P = 0.0001) but not by protein consumed.

Similar results were found when larvae fed on S++ and A genotypes. Second instar larvae on A consumed more protein (1.3 g) than those on S++ (0.8 g) plants, but no differences were found during the first and third instars (F_1,49-Second _= 152.9; P < 0.0001; F_1,49-Third _= 1.0; P = 0.3; Fig. 5/Appendix 1). The calculated total amount of protein consumed was higher for larvae on A (7.0 g) than on S++ (6.3 g) genotypes (F_1,49-Total _= 7.8; P = 0.007; Fig. 5/Appendix 1). Larval mass of caterpillars on A and S++ genotypes was affected by the amount of TPI and protein consumed (F_1,49-11d _= 49.8; P < 0.0001). In summary, caterpillar fed on high TPI-genotypes consumed more TPI and less protein than those larvae fed on low TPI-genotypes.

**Figure 6:**
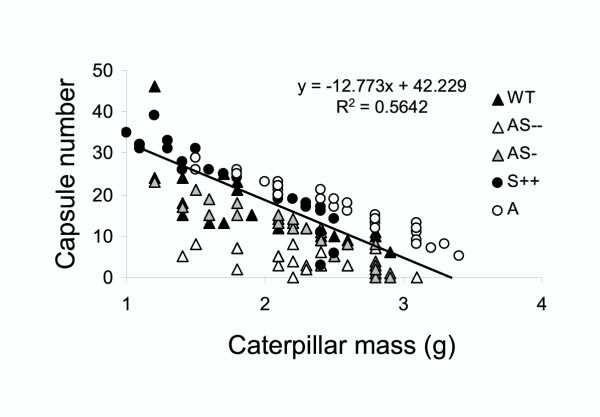
Seed capsule production per plant of *N. attenuata *genotypes (WT, AS –, AS-, S++ and A), regressed against *M. sexta *larvae mass (g) 11 d after neonates started to feed on the leaf at S1 position (elongation stage). Line represents a regression fitted to the points (Y = -12.773 (g) + 42.229; R^2 ^= 0.5642).

We estimated the effect of the differences in larval movement by simulating TPI and protein consumption by transposing movement and consumption patterns from untransformed (WT and A) to transformed (AS –, AS-, and S++) plants as explained in the supplemental section (Fig. 6 and Tables 1a and b/Appendix 1). Patterns of larval movement on WT plants (S_WT_) did not alter TPI consumed on the AS (AS – and AS-) genotypes when WT movement data were transposed to larvae on AS genotypes (P_AS – _= 0.9; P_AS- _= 0.09); the highest values were found in the calculated WT genotype (F_4,126 _= 545.5; P < 0.0001; Fig. 6/Appendix 1). WT daily movement patterns decreased S_WT _protein consumed from AS – genotype plants (F_4,126 _= 11.7; P < 0.0001; Fig. 6/Appendix 1). Larval movement on AS – plants increased TPI and protein consumed on WT plants (F_4,127-TPI _= 473.8; P < 0.0001; F_4,127-Protein _= 8.1; P < 0.0001; Fig. 6/Appendix 1).

Larval movement on A plants did not change the amount of TPI consumed on S++ genotype plants (F_1,49 _= 1.5; P = 0.2) but did increase the amount of protein consumed (F_2,74 _= 7.4; P = 0.001; Fig. 6/Appendix 1); larval movement on S++ plants did not change protein consumed on A genotype (F_2,73 _= 3.8; P = 0.2; Fig. 6/Appendix 1). In summary, when larval movement patterns on low TPI plants were transposed to high TPI genotypes, protein and TPI consumption increased. Transposing WT movement patterns to AS – genotype decreased the amount of protein consumed.

### Fitness consequences of TPI expression for plants attacked by M. sexta larvae

To determine whether expression of TPIs increases *N. attenuata'*s fitness when plants are attacked by *M. sexta *larvae, we measured caterpillar mass on and capsule number per plant from transformed and untransformed genotypes with either low or no TPI activity (A, AS –, and AS-) and high TPI activity (WT and S++). Larval mass of caterpillars fed on low TPI genotypes were higher (45-21 %) than those fed on genotypes with high TPI activity (F_4,35-4d _= 20.0; P < 0.0001; F_4,195-11d _= 8.6; P < 0.0001; Fig. 4), those that fed on either WT or S++ (F_1,14-4d _= 0.02; P = 0.9; F_1,78-11d _= 0.1; P = 0.6) or AS – or A (F_1,14-4d _= 0.01; P = 0.9; F_1,78-11d _= 1.6; P = 0.2) did not differ (Fig. 4).

We measured lifetime seed capsule number per plant on unattacked and attacked plants and calculated the mean differences and the percentage mean differences between treatments in order to estimate fitness consequences of constitutive and inducible TPI production. As expected, mean capsule number in unattacked plants was higher on genotypes with either low or no TPI activity (A and AS –) than on genotypes with intermediate and high TPI activity (WT, S++, and AS-; Fig. 5), which reflects the fitness cost of TPI production. Eleven days of caterpillar attack reduced seed capsule production per plant in all genotypes and reversed the pattern of seed capsule production among high and low TPI-containing genotypes. Within the group of transformed (AS – and AS-) and untransformed (WT) unattacked plants from the Utah genotype, mean capsule number was higher (22–25 %) on the genotype with low TPI activity (AS –) than on genotypes with intermediate and high TPI activity (AS-and WT; F_2,81 _= 8.6; P = 0.004; Fig. 5); however after 11 d of caterpillar attack, mean capsule number, absolute and relative mean difference in capsule number were the highest on WT (15 capsules) and the lowest on AS – (4 capsules) genotypes (F_2,81 _= 25.3; P < 0.0001; Fig. 5 and Table 1). Within the Arizona genotypes, mean capsule number of unattacked plants was higher on the genotype with no TPI activity (A; 49 capsules) than on the genotype with high TPI activity (S++; 35 capsules; F_1,54 _= 16.4; P = 0.0002; Fig. 5). However, when plants were attacked, mean capsule number as well as absolute and relative mean difference in capsule number were higher on S++ (23 capsules) than on A genotypes (17 capsules; F_1,54 _= 7.9; P = 0.006; Fig. 5 and Table 1).

**Table 1:** Absolute and relative mean differences between treatments in seed capsule production and TPI levels from either untransformed wildtype (WT) or homozygous T_3 _independently transformed lines of a WT genotype of *Nicotiana attenuata *which had been transformed with constructs containing the *pi *gene in an anti-sense orientation (AS –, AS-); absolute and relative mean differences between untransformed plants of the Arizona (A) genotype and plants of the Arizona genotype transformed with constructs containing the full-length *pi *gene in a sense (S++) orientation, that were either unattacked or attacked by *Manduca sexta *larvae for 11 days.

**Genotypes**	**Mean diff. in capsule number**	**% Mean diff. in capsule number**	**P**	**TPI levels**
WT	18.04	54.18	<0.0001	High
AS--	40.36	91.35	<0.0001	Low
AS-	23.14	68.14	<0.0001	Intermediate
S++	12.64	35.47	<0.0001	High
A	32.25	65.58	<0.0001	No TPI

P-values are from one-way ANOVAs between treatments.

In order to determine the effect of caterpillar attack on seed capsule production per plant, we regressed caterpillar mass against seed capsule production per plant from transformed and untransformed genotypes and found that a linear equation (Y = -12.7 (g) + 42.2; R^2 ^= 0.5; Fig. 6; P < 0.0001) represented the best fit. The relationship suggests that the higher the *M. sexta *larvae mass, the lower the seed capsule number production per plant.

## Discussion

Our experiments demonstrate that the benefits of TPI expression in *N. attenuata *grown in greenhouse conditions outweigh their costs when plants are attacked by *M. sexta *larvae. Unattacked plants with low constitutive TPI levels produced more seed capsules (AS–: 44, AS-: 34 and A: 49 capsules) than did plants with high TPI levels (WT: 33 and S++: 35 capsules), and 11 days of *M. sexta *attack reduced seed capsule production per plant in all genotypes and reversed the pattern of seed capsule production with higher reductions in AS (AS–: 91 % and AS-: 68 %) and A (65 %) than in WT (54 %) and S++ (35 %) plants (Fig. 5 and Table 1). This differential reduction in seed capsule production amongst genotypes correlated negatively with larval mass. Across all genotypes, the larger the larval mass, the lower the number of capsules per plant (Fig. 6). This result is consistent with previous demonstrations that endogenous TPIs decrease the performance of *M. sexta *[33] and with the central prediction of the Optimal Defense theory, namely that defense is costly [23,35,37]. Moreover, the results highlight the heuristic value of the cost-benefit paradigm for functional studies. However, conclusive evidence that TPI expression in *N. attenuata *outweigh their costs when plants are attacked will require field experiments in which both ecological and allocation costs of defense can arise. For example, constitutive and inducible TPI production incurs large fitness costs in *N. attenuata *when plants where grown with competitors [32,42], one of the dominant selective factors for this species [30]. In addition, other factors such as temperature and *M. sexta *predators can affect feeding damage [38].

Despite the central role of the cost-benefit model of inducible defenses, the vast majority of research in this area examines how inducible defenses influence either herbivore performance or plant fitness in separate experiments and their currencies are usually not comparable (i.e., plant fitness for the cost; herbivore performance for the benefits). Few studies have tested the cost-benefit model by measuring both costs and benefits in the same currency (plant fitness for both the costs and benefits) and have elicted plant defenses by either applying methyl jasmonate or damaging leaves [5,11]. However, because of the pleiotorpic effects of the elicitors, the observed fitness differences do not arise solely from the expression of the resistant trait [30,43], and therefore these studies are likely to overestimate the fitness cost of resistance. Direct genetic manipulation of TPI expression allowed us measure the costs and benefits of a defensive protein in a plant-insect interaction in the common currency of plant fitness.

Transformation technology gave us the means to manipulate TPI expression with high precision. Antisense expression of the *pi *gene reduced constitutive and caterpillar induced TPI levels in AS – and AS-genotypes (by 35–80% of the activity of WT) in S1 and stem leaves without influencing protein contents. Caterpillar attack increased TPI levels 2–2.5-fold in either WT or AS genotypes (Fig. 2; Figs 1-3/Appendix 1) but the absolute levels were substantially lower in the AS genotypes. Transformation of the A genotype with a functional TPI gene under the control of a constitutive promoter (S++ genotype) produced TPI levels that were 74% of the activity found in caterpillar attacked WT plants (Fig. 2; Fig. 4/Appendix 1). Because these transformed lines did not differ in any other measured defense traits [42], they allowed us to examine the defensive function of TPIs by constraining plant responses to herbivore attack and observe unconstrained herbivore behavior in response to these constrained plant responses. In this way, the dynamics of the plant responses, or the lack thereof, are reflected in the herbivore behavior. Low constitutive TPI expression in the host plant may increase proteolytic enzyme activity in the guts of neonates, digestion efficiency and the growth rates [44] (Fig. 4). This early increase in larval growth rate translates into increases in pupal mass, which is an accurate proxy for fecundity in Lepidoptera [45,46], but may also profoundly influence larval movement.

Given the large within-plant heterogeneity in food quality, it is reasonable to expect a complex resource-oriented larval behavior that changes with instars [27,47]. Moving has been shown to be costly during the first 3 instars [26,38], but these costs are thought to decrease with size [48]. Larvae with larger mass (on either low or no TPI-producing genotypes) left the S1 leaf 1–2 days earlier than did those with lower mass (on high TPI-producing genotypes; Fig. 3). The heavier larvae moved earlier than lighter larvae to young leaves which typically have higher levels of protein and water contents [27,48,49]. Based on the calculations, larvae fed on high TPI genotypes consumed 3–4 fold more TPI and 10 % less protein than did larvae feeding low TPI genotypes over the 11d of the experiment (Fig. 3 and 4; Fig. 5/Appendix 1). Since we did not measure the amount of leaf consumed by larvae and the values used for the calculation of protein consumption are from plants with natural TPI levels, the calculations likely underestimate the amount of protein consumed. These results suggest that a high TPI content keeps caterpillars from feeding on the high-protein younger leaves at the TOP of the plants possibly by decreasing larval mass and thereby their ability to move. Larval movement influences the caterpillar's ability to compensate for variation in diet quality.

By moving, caterpillars can exploit the high within-plant heterogeneity in food quality to compensate for nutritional imbalances. For example, *Helicoverpa zea *larvae feed on multiple plant structures to balance their amino acid requirements [50]. *M. sexta *larvae fed low protein and nutritionally unbalanced diets compensated not only for the decreased protein intake [51] but also for unbalanced nutrition by selecting diets high in the missing nutrients which increased larval growth rates [52,53]. Growth depends on nutrient ratios, and insects may use behavioral and post-ingestive mechanisms to compensate for nutrient imbalances [54,55]. To estimate the consequence of higher caterpillar mass on movement, we transposed the larval location data of caterpillars from those observed on low-to high-TPI genotypes, and found increased larval protein (10 %) and TPI (12 %) consumption (Fig. 6/Appendix 1). Transposing daily larval location data in the opposite direction decreased (by 10 %) protein consumed but did not influence TPI consumption (Fig. 6/Appendix 1). These calcuations suggest that *M. sexta *caterpillars may adjust their feeding positions to minimize TPI consumption and maximize protein intake. Hence the naturally occurring high TPI levels delay larval growth and prevent caterpillars from feeding on high-quality younger leaves, which may have a high fitness value for the plant [35,50,56].

The interaction between *N. attenuata *and *M. sexta *starts with moths ovipositing on leaves at the bottom of the plant; oviposition is influenced by temperature, food quality and quantity, and predation risk [38]. Plants respond by increasing TPI levels, which decreases larval mass and survivorship [33], and by increasing the emission of volatile organic compounds, which alters oviposition choices and attracts the generalist predator *Geocoris pallens *to feeding larvae [31]. *Geocoris *is size selective and preferentially attacks eggs and larvae in the first three instars. The up-regulation of TPIs by herbivore attack slows larval growth and keeps larvae in stages that are more vulnerable to the predator, thus increasing larval mortality [57]. Interestingly, the volatile signals that function as indirect defenses by attracting *Geocoris *to feeding larvae are elicited by the same signals that elicit TPI production [34,36,58], providing the mechanism of coordination among these defense system.

Once larvae reach a mass that can compensate for the cost of movement, they leave the leaf with high TPI levels and move upward within the host plant and feed preferentially on young leaves with high levels of protein and nicotine, which increases larval mass and decreases plant fitness [35,38,51]. A starvation period during the firsts instars was found to reduce *M. sexta *larval development more than feeding on fully JA-induced (high TPIs) *N. attenuata *leaves [40]. Thus for these larval instars, the costs of movement, which include increases in starvation and predation risks are likely greater than the costs of coping with a plant's induced defenses. Other generalist herbivores on *N. attenuata*, namely noctuid larvae and weevil beetles, usually attack older leaves that are lower in nutrients as well as nicotine [30,35,38]. Nicotine is not an efficient defense against *M. sexta*, because this insect is adapted to feed on *N. attenuata *and larvae can detoxify nicotine [59-61]. Moreover, its attack down-regulates nicotine production which could be sequestered by the herbivore and maybe co-opted and used as a defense against parasitoids [30,62,63]. Hence the plant relies on other defenses when attacked by *M. sexta *larvae: TPIs, for example, decrease larval mass and prevent caterpillars from feeding on leaves with high fitness value for the plant. This delayed in caterpillar movement upward within the plant, maybe a result of larvae adaptation to high leaf-TPI levels by increasing the production of insensitive gut proteases to TPIs [29]. Eliciting only those defenses that confer resistance to the attacking herbivore (targeting), rather than the entire defensive repertoire, may minimize the cost of resistance [14].

## Conclusions

We conclude that despite the ongoing evolutionary interaction between *N. attenuata *and *M. sexta*, TPI-mediated decreases in herbivore performance translates into a fitness benefit for the plant.

## Methods

### Plant material and transformation

*N. attenuata *used in this study were grown from seeds collected from either Utah [5] or Arizona [32] and inbred 10 and 4 generations, respectively. In order to silence the expression of *N. attenuata*'s *pi *gene in the genotype collected in Utah (WT), WT was transformed by an *Agrobacterium*-mediated transformation procedure with pNATPI1, which contains 175 bp of *N. attenuata*'s 7-repeat domain *pi *gene in an anti-sense orientation (AS), as described in [32]. Southern gel blot analysis confirmed that all T_3 _lines were single-copy independent transformants [42].

In this study, we used a genotype of *N. attenuata *collected from Arizona (A), with methyl jamonate (MeJA)-inducible nicotine levels identical to that found in WT plants, but completely lacking the ability to produce TPIs or accumulate TPI mRNA [32]. More recently, the mutation in the 7-domain repeat *pi *of A plants has been characterized and found to be located in the 5'signal peptide, resulting in a premature stop codon (J. Wu and I.T. Baldwin, unpublished data). Because we never detected TPI activity with radial diffusion assay in A genotype [34], nor have we detected TPI mRNA transcript with either northern blot analysis or reverse transcriptase-PCR, we suggest that this transcript is rapidly silenced [33]. Plants of the A genotype were transformed with a binary transformation vector pRESC2PIA2 containing the full-length 7-domain *N. attenuata pi *gene from the WT genotype in the sense orientation under control of the constitutive CaMV 35S promotor [42]. Several T_3 _lines harboring a single copy of the transgene [42] were screened for TPI activity, and all had TPI activity comparable to that of elicited WT plants. One of these A lines (S++) with 60% of the activity of MeJA-elicited WT plants was selected for study. Arizona non-transformed plants (A) had no detectable TPI activity. All of these transformed and untransformed genotypes were used in the experiments and the quality of the seeds that these genotypes produce do not differ from the seed quality of the WT.

### Bioassay experiments and plant fitness determination

In order to determine the effect of *M. sexta *herbivory on the fitness of *N. attenuata*'s genotypes using either down-regulation or restored expression of the *pi *gene, a single *M. sexta *neonate was placed on the leaf growing at node S1 (Fig. 1) of 48 soil-grown plants in elongation stage of AS lines (AS – and AS-), on A line transformed to express the functional *pi *(S++), and on untransformed genotypes (WT and A). Larvae were allowed to move and feed freely on plants for 11 days. Their mass was determined 4 and 11 days after hatching. Larval movement on the plant during this time was monitored, and larval location on the plant classified as follows: S1 (leaf where larvae started to feed), BOTTOM (from 0 to S3 leaf position), MIDDLE (from S4 to S5 leaf position), and TOP (from S6 to S9 leaf position; Fig. 1). Eggs of *Manduca sexta *L. (Lepidoptera: Sphingidae) were obtained from Carolina Biological Supply Company (Burlington, North Carolina, USA) and placed in plastic containers (200 mL) on a moist tissue. The containers were kept in climate chambers at 28°C and 65 % relative humidity under a 16:8 h light:dark photoperiod until the eggs hatched.

Seeds were germinated in diluted liquid smoke solutions as described in [64]. Seedlings were transplanted in 1-L pots in a glasshouse under the conditions described in [42] with 1000 – 1300 μmol m^-2 ^s^-1 ^PPFD supplied by 450 W Na-vapor HID bulbs. To compare the lifetime reproductive performance among genotypes after being either unattacked or attacked by *M. sexta *larvae, we recorded the number of seed capsules per plant from 28 plants (8 + 12 plants were used to TPI determination) of each genotype and treatment combination two weeks after last watering day. Daily watering stopped 21 d after neonates started to feed on the leaf, in order to mimic the drying and termination of growth in the plant's natural habitat, the Great Basin Desert. The number of capsules per plant reflects the lifetime reproductive output (seeds) in *N. attenuata *under natural or glasshouse conditions [5,65].

Constitutive and TPI activity induced by caterpillar damage were determined from stem and rosette leaves before the larvae were placed on the leaf at node S1 (8 plants; 4 rosette leaves; 5 stem leaves; Fig. 1), and 4 (8 plants; 4 rosette leaves; 8 stem leaves) and 11 (12 plants; 4 rosette leaves; 14 stem leaves) days after the larvae started to feed. During the last harvest TPI activity was also determined on axillary leaves from S1 to S4 nodes. Protein concentrations and TPI activity were measured and expressed as nmol mg^-1 ^as described in [34]. Larvae TPI and protein consumption were calculated and simulated as explained in the supplemental section.

### Statistical analysis

Data were analyzed with Stat View, Version 5.0 (SAS, 1998). The TPI, protein and larval mass, and calculated and simulated values were analyzed by ANOVAs followed by Fisher's protected LSD *post-hoc *comparisons in all experiments. Differences in larval number on plants were analyzed with the Mann-Whitney *U*-test.

## Author's contributions

JAZ carried out the experiments and analyzed the data, while planning of the experiment and writing of the manuscript was a joint effort by JAZ and ITB.

## Supplementary Material

Additional File 1 Calculation of TPI and protein consumed by *M. sexta *larvae. Table 1. Combination of TPI and protein from different *N. attenuata *genotypes and larval location of different genotypes that have either calculated (C) or simulated (S) values. Fig. 1: TPI activity (mean ± SEM) and protein content from different leaf positions of WT plants at the elongation stage. Fig. 2: TPI activity (mean ± SEM) and protein content from different leaf positions of AS – plants at the elongation stage. Fig. 3: TPI activity (mean ± SEM) and protein content from different leaf positions of AS-plants at the elongation stage. Fig. 4: TPI activity (mean ± SEM) and protein content from different leaf positions of S++ plants at the elongation stage. Fig. 5: Calculated TPI and protein consumed by *M. sexta *larvae fed on WT, AS–, AS-, S++, and A genotypes during the first, second and third instars. Fig. 6: Calculated and simulated TPI and protein consumed by *M. sexta *larvae. Click here for file
