# Supplementary material for: Fitness benefits of trypsin proteinase inhibitor expression in Nicotiana attenuata are greater than their costs when plants are attacked
Source: BMC Ecol. 2004 Aug 10;4:11. doi: 10.1186/1472-6785-4-11 (PMC514560; doi:10.1186/1472-6785-4-11)
Supplement: Additional File 1 — Calculation of TPI and protein consumed by M. sexta larvae. Table 1. Combination of TPI and protein from different N. attenuata genotypes and larval location of different genotypes that have either calculated (C) or simulated (S) values. Fig. 1: TPI activity (mean ± SEM) and protein content from different leaf positions of WT plants at the elongation stage. Fig. 2: TPI activity (mean ± SEM) and protein content from different leaf positions of AS – plants at the elongation stage. Fig. 3: TPI activity (mean ± SEM) and protein content from different leaf positions of AS-plants at the elongation stage. Fig. 4: TPI activity (mean ± SEM) and protein content from different leaf positions of S++ plants at the elongation stage. Fig. 5: Calculated TPI and protein consumed by M. sexta larvae fed on WT, AS–, AS-, S++, and A genotypes during the first, second and third instars. Fig. 6: Calculated and simulated TPI and protein consumed by M. sexta larvae. [file 1472-6785-4-11-S1.DOC]

**Appendix 1.**

**Calculation of TPI and protein consumed by *M. sexta* larvae.**

In order to determine the amount of TPI and protein consumed by *M. sexta* larvae during the experiment, we estimated leaf TPI and protein values for days 1, 2, and 3 with daily leaf concentrations extrapolated from measurements at day 0 and day 4, and for days 5 - 10 with measurements at day 4 and day 11 for all rosette and stem leaf positions (Supplemental Figures 1-4). With these concentration values, the location of the larvae each day on the plant (Figs 1 and 3), and information from the literature about the average leaf area consumed by a *M. sexta* larvae during the first, second, and third instars [1], we calculated the amount of TPI and protein consumed (C) for larvae feeding on each genotype (Additional data file Fig. 5). These calculated values are products of the larvae’s instar-specific feeding site, amount, and TPI and protein content of each leaf position consumed (Additional data file Tables 1a and b). Calculated values were obtained as follows: protein consumed = leaf protein content (mg/g) x larvae consumption (g) and for TPI consumed = leaf TPI content (nmol/g) x larvae consumption (g).

In order to determine the effect of larval movement on TPI and protein consumption, we simulated larval TPI and protein consumption on transformed (S_T_) and untransformed (S_WT_ and S_A_) genotypes by transposing either daily larval location data (S_WT_ and S_A_) or leaf TPI and protein content (S_T_) from untransformed (WT and A) to transformed (AS--, AS-, and S++) genotypes (Additional data file Tables 1a and b). For example, to calculate the S_T_ value in combination with WT either TPI or protein content and larvae location on AS-- genotype, we used the TPI and protein contents of the WT and calculated the TPI and protein consumption using the larval location on AS-- plants (Additional data file Table 1a).

**Additional data file Table 1.** Combination of TPI and protein from different *N. attenuata* genotypes and larval location of different genotypes that have either calculated (C) or simulated (S) values. **a**. WT, AS-- and AS- genotypes. **b.** S++ and A genotypes. C values resulted from larval location and either TPI or protein from the same genotype. S_WT_ and S_A_ values resulted from transposing larval location data from untransformed (WT and A) to transformed (AS--, AS-, and S++) genotypes. S_T_ values resulted from transposing either TPI or protein contents from untransformed (WT and A) to transformed (AS--, AS- and S++) genotypes.

TPI and protein from genotypes

|  | **a** | **WT** | **AS--** | **AS-** |  | **b** | **S++** | **A** |
| --- | --- | --- | --- | --- | --- | --- | --- | --- |
| Larval | **WT** | C | S_WT_ | S_WT_ |  | **S++** | C | S_T_ |
| location | **AS--** | S_T_ | C |  |  | **A** | S_A_ | C |
|  | **AS-** | S_T_ |  | C |  |  |  |  |

**References**

1. AH Madden, FS Chamberlin: **Biology of the tobacco horn-worm in the southern cigar-tobacco district.** *USDA Tech Bull* 1945, **986**:1-51.


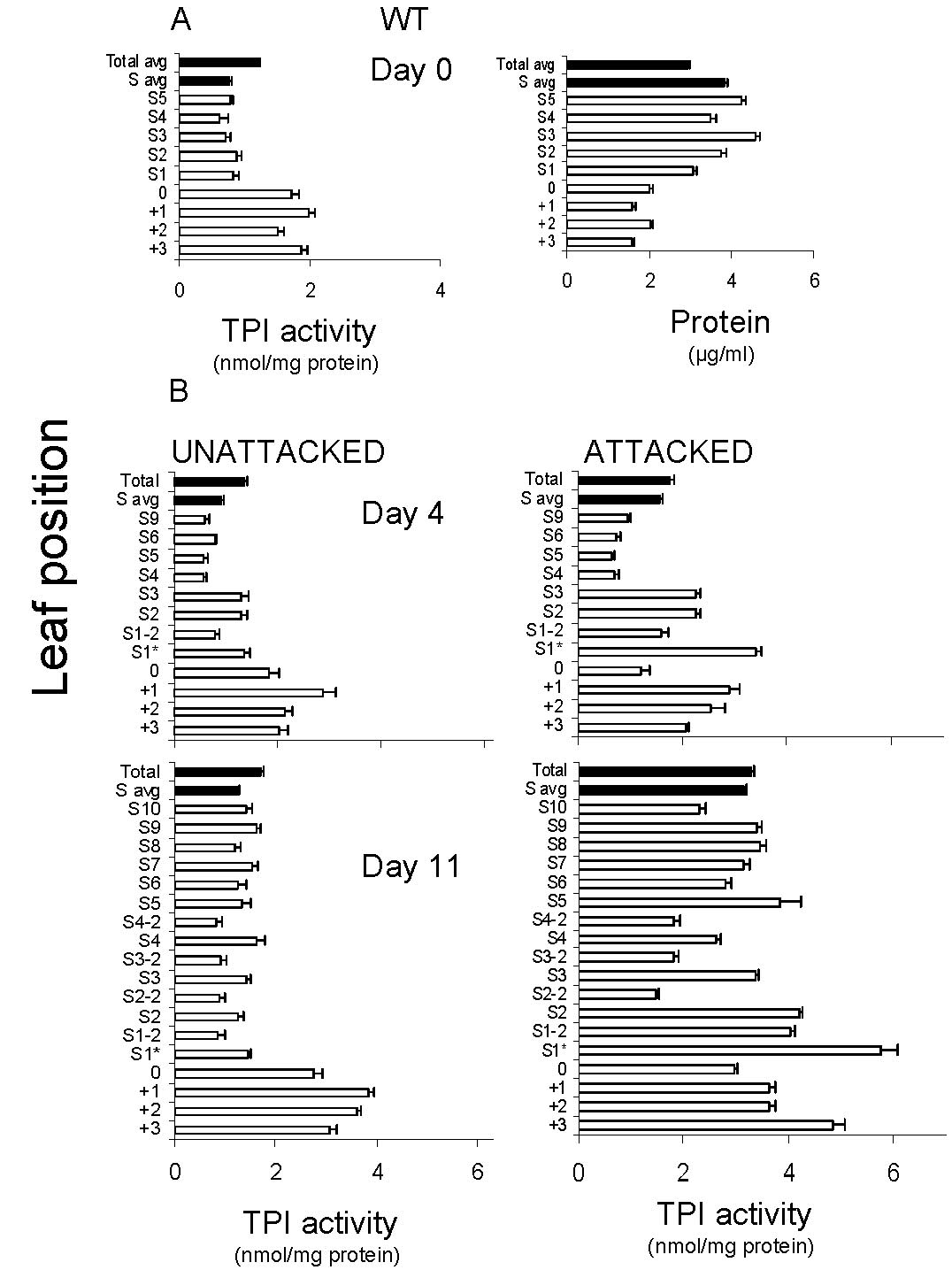


**Additional data file Figure 1. A.** TPI activity (mean ± SEM) and protein content from different leaf positions of WT plants at the elongation stage before *M. sexta* larvae attack. **B.** TPI activity (mean ± SEM) from different leaf positions of the WT plants at the elongation stage either unattacked or attacked by *M. sexta* larvae 4 and 11 d after neonates started to feed on the leaf at S1 position (*). Solid bars show the average of TPI activity and protein content either of rosette and stem leaves (Total avg) or of stem leaves only (S avg). See Figure 1 for graphic depiction of leaf position.


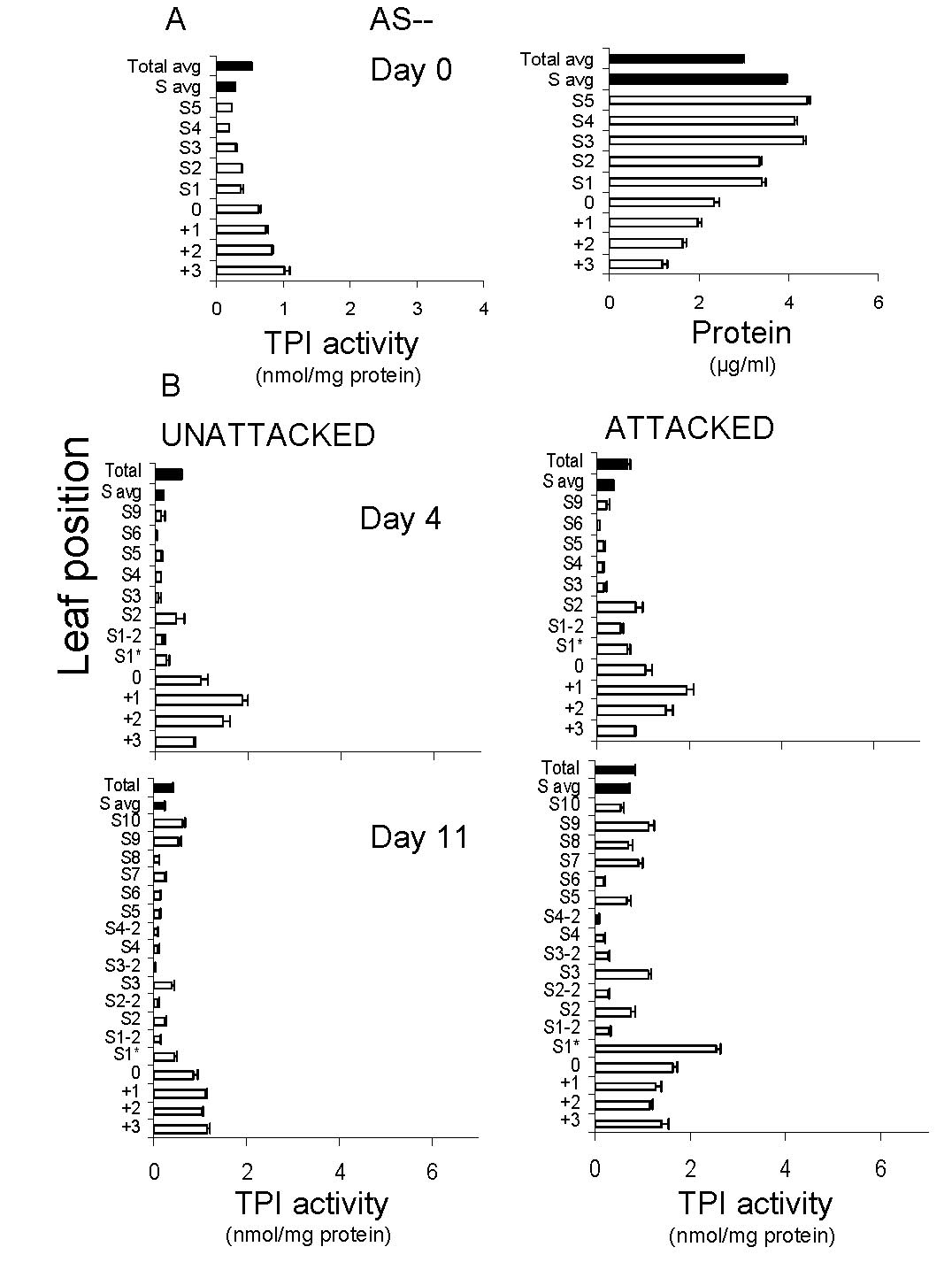


**Additional data file Figure 2. A.** TPI activity (mean ± SEM) and protein content from different leaf positions of AS-- plants at the elongation stage before *M. sexta* larvae attack. **B.** TPI activity (mean ± SEM) from different leaf positions of the AS-- plants at the elongation stage either unattacked or attacked by *M. sexta* larvae 4 and 11 d after neonates started to feed on the leaf at S1 position (*). Solid bars show the average of TPI activity and protein content either of rosette and stem leaves (Total avg) or of stem leaves only (S avg). See Figure 1 for graphic depiction of leaf position.


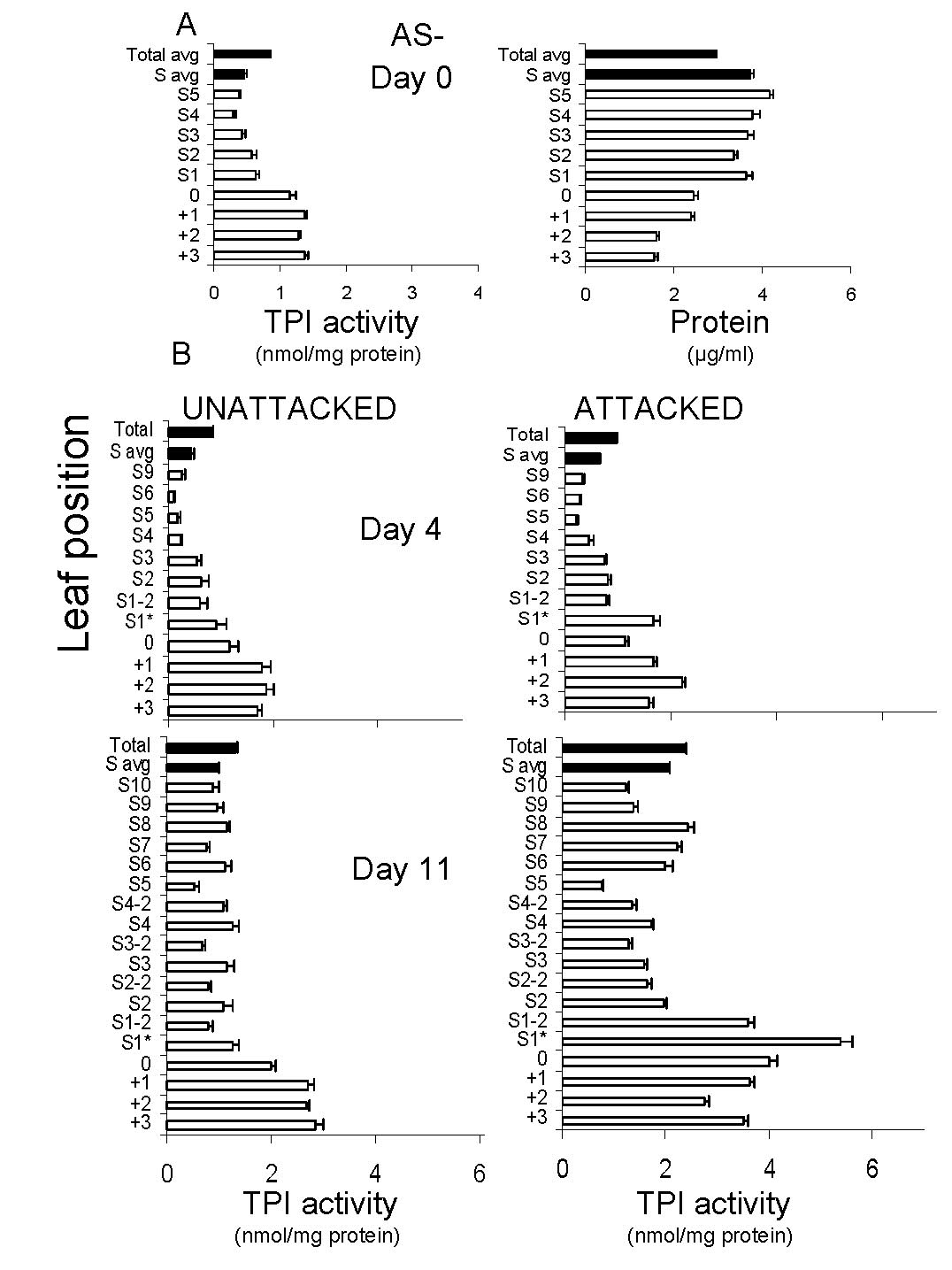


**Additional data file Figure 3. A.** TPI activity (mean ± SEM) and protein content from different leaf positions of AS- plants at the elongation stage before *M. sexta* larvae attack. **B.** TPI activity (mean ± SEM) from different leaf positions of the AS- plants at the elongation stage either unattacked or attacked by *M. sexta* larvae 4 and 11 d after neonates started to feed on the leaf at S1 position (*). Solid bars show the average of TPI activity and protein content either of rosette and stem leaves (Total avg) or of stem leaves only (S avg). See Figure 1 for graphic depiction of leaf position.


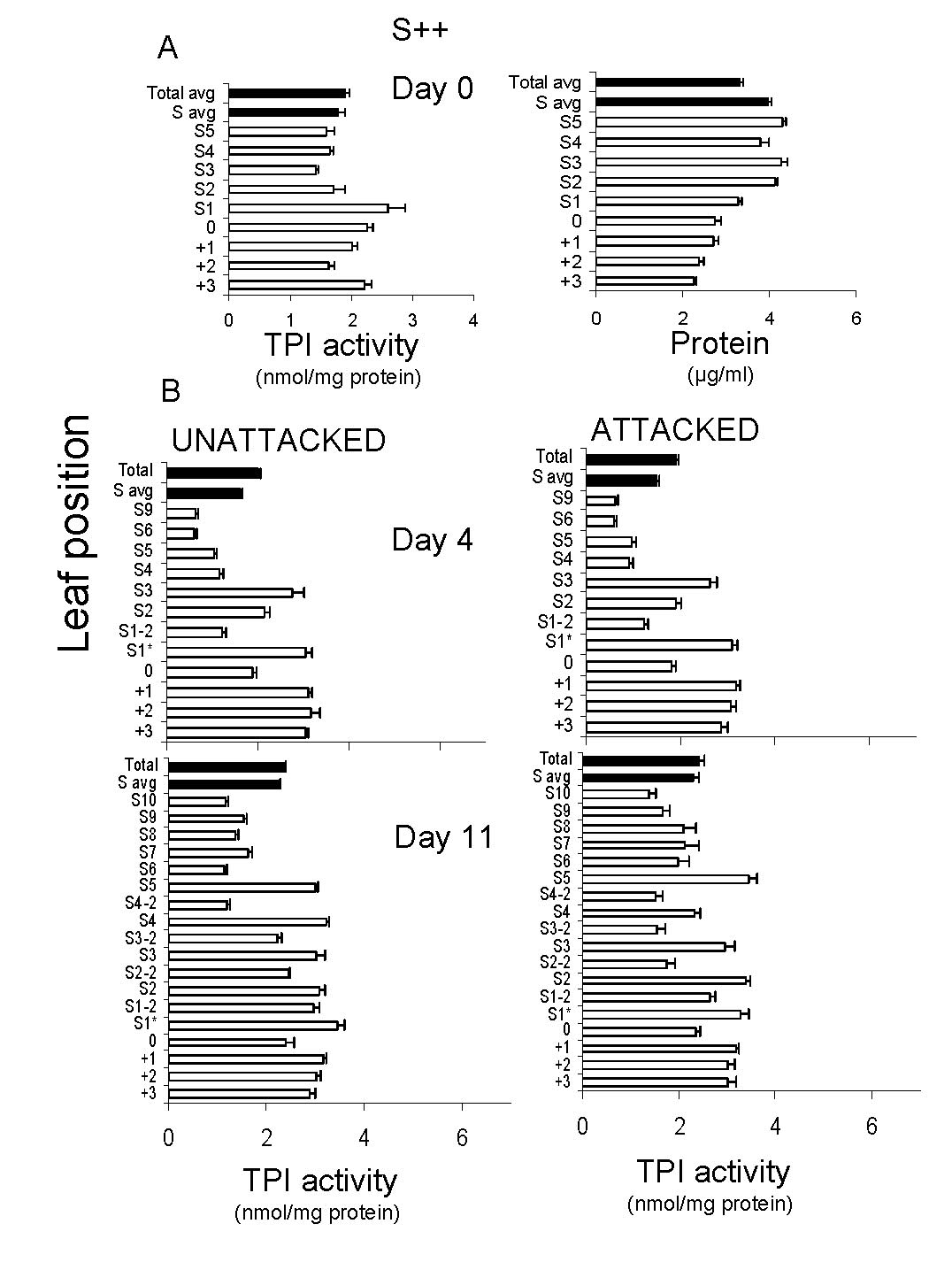


**Additional data file Figure 4. A.** TPI activity (mean ± SEM) and protein content from different leaf positions of S++ plants at the elongation stage before *M. sexta* larvae attack. **B.** TPI activity (mean ± SEM) from different leaf positions of the S++ plants at the elongation stage either unattacked or attacked by *M. sexta* larvae 4 and 11 d after neonates started to feed on the leaf at S1 position (*). Solid bars show the average of TPI activity and protein content either of rosette and stem leaves (Total avg) or of stem leaves only (S avg). See Figure 1 for graphic depiction of leaf position.


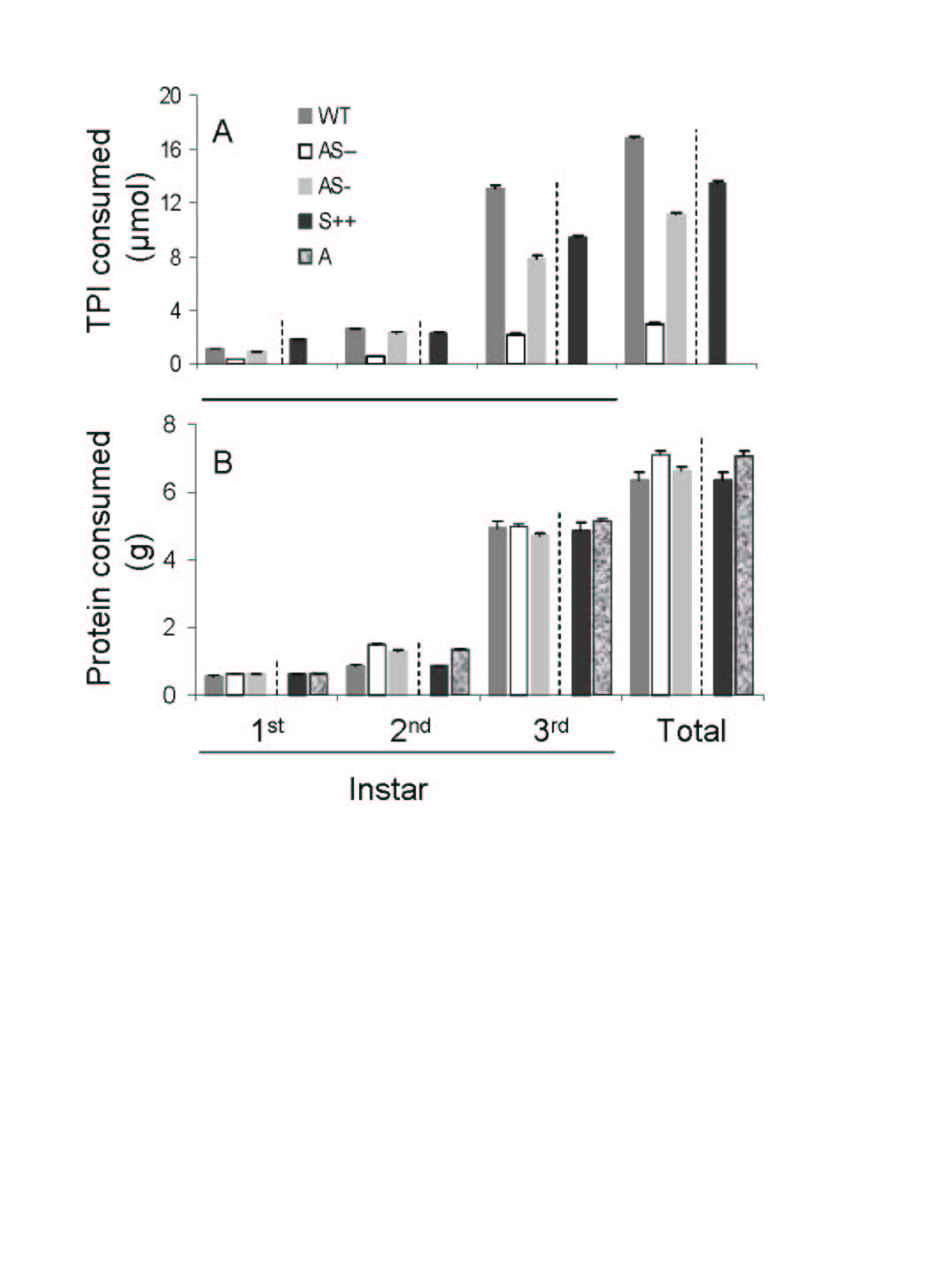


**Additional data file Figure 5.** Calculated TPI and protein consumed by *M. sexta* larvae fed on WT, AS--, AS-, S++, and A genotypes during the first, second and third instars. These calculated values are products of larvae’s instar-specific feeding site, amount, and TPI and protein content of each leaf position consumed and are extrapolated from measurements from days 0 and 11.

**
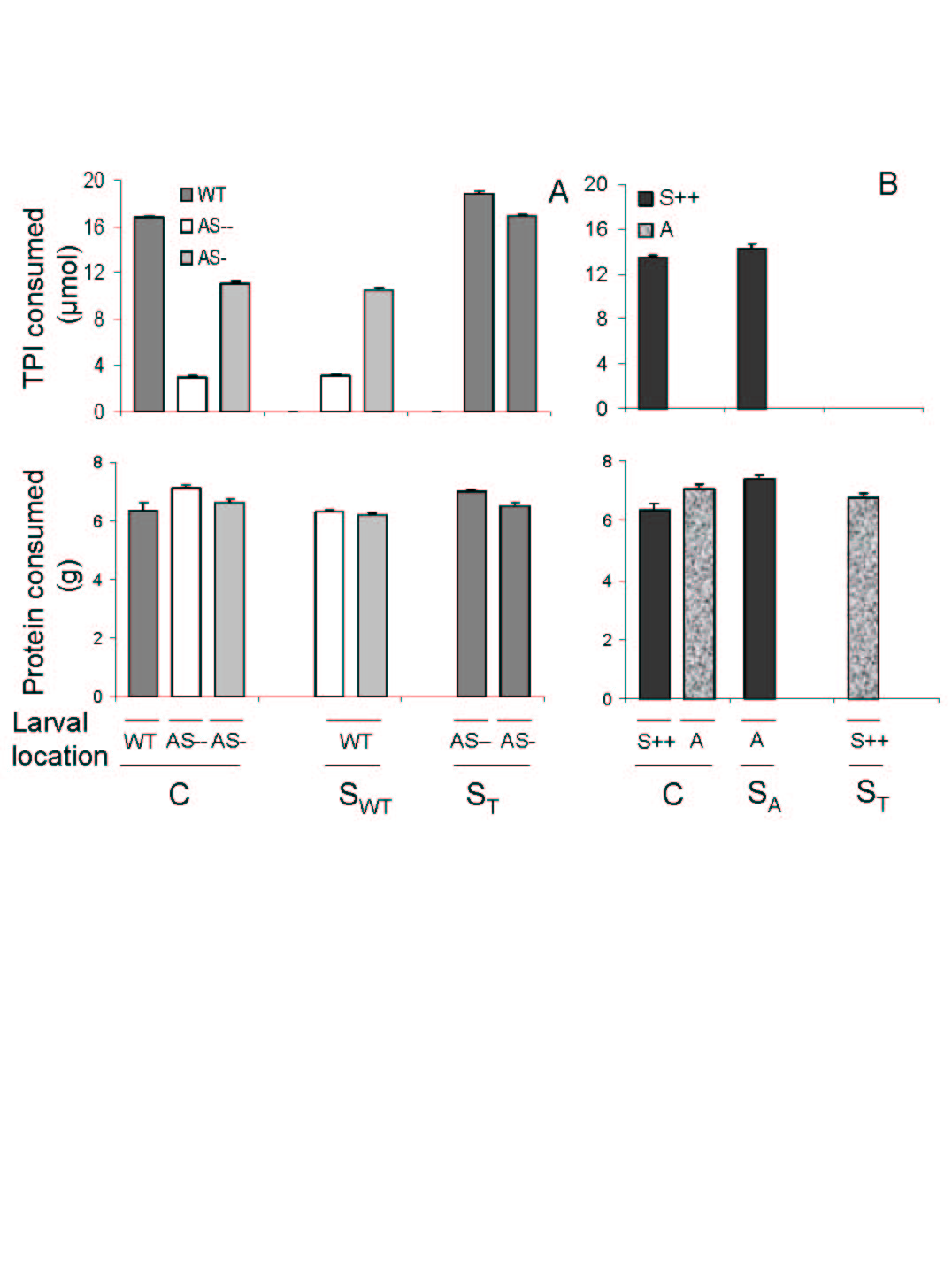
 Additional data file Figure 6.** Calculated (C) and simulated (S) TPI and protein consumed by *M. sexta* larvae. **A.** Genotypes and larval location of WT, AS--, and AS- . **B.** Genotypes and larval location of S++ and A. C values resulted from larval location and either TPI or protein from the same genotype. S_WT_ and S_A_ values resulted from transposing larval location data from untransformed (WT and A) to transformed (AS--, AS-, and S++) genotypes. S_T_ values resulted from transposing either TPI or protein contents from untransformed (WT and A) to transformed (AS--, AS- and S++) genotypes (Supplemental Tables 1a and b).
